# Ultrasound-Guided Modified Seldinger Placement of Tenckhoff Catheters in Pediatric Patients Undergoing Peritoneal Dialysis: Single Center Experience

**DOI:** 10.3389/fped.2022.917720

**Published:** 2022-06-30

**Authors:** Yang Yu, Qing Xie, Yaxian Chen, Wanmei Hu, Panpan Zhang, Shi Huang, Fengjie Yang, Yonghua He, Yonghong Yi, Jianhua Zhou, Yu Zhang

**Affiliations:** ^1^Department of Medical Ultrasound, Tongji Hospital, Tongji Medical College, Huazhong University of Science and Technology, Wuhan, China; ^2^Department of Pediatrics, Tongji Hospital, Tongji Medical College, Huazhong University of Science and Technology, Wuhan, China

**Keywords:** PD catheter, percutaneous insertion, modified Seldinger technique, children PD catheter, children

## Abstract

Minimally invasive peritoneal dialysis (PD) catheterization is increasingly common, and percutaneous PD catheters may be placed using a trocar or the Seldinger technique. There are few reports of pediatric percutaneous PD catheter insertion. We retrospectively compared the outcomes from percutaneous placement of Tenckhoff catheters using a modified Seldinger technique with catheter placement by open surgery. This single-center retrospective study compared 14 pediatric patients who received percutaneous PD catheter insertion using an ultrasound-guided modified Seldinger technique (August 2018–February 2021) with 10 patients who received open-surgical PD catheter insertion (2015–2018). Complications and catheter survival were evaluated. The overall technical success rate was 100%, but the Seldinger technique required less time (30 vs. 45 min) and smaller incisions (1.1 vs. 4.4 cm). The early complications in the Seldinger and control groups were bleeding (1 vs. 0), catheter dysfunction (1 vs. 1), abdominal pain (3 vs. 7), and exit leakage (0 vs. 1). In the Seldinger group, the median time from insertion to first use was 3 days, and the minimum follow-up was 6 months. Catheter survival at 6 months was 93% (Seldinger group) and 90% (open surgery group). The adoption of this technique at our institution led to a significant increase in the percentage of new pediatric dialysis patients commencing PD rather than hemodialysis. Collectively, the modified Seldinger technique described here was safe and feasible in pediatric patients. This approach is simpler and more rapid than open surgery, and reduces early complications and increases PD uptake.

## Introduction

Peritoneal dialysis (PD) is the most widely used initial renal replacement treatment modality worldwide for children with end-stage renal disease (1). Of pediatric patients requiring long-term dialysis, the proportion receiving PD is estimated to be 50–70% in developed countries and likely considerably higher in developing countries. PD provides children with several advantages over hemodialysis (HD), including the ability to regularly attend school and engage in normal childhood activities, a reduced need for dietary restrictions, and better preservation of residual renal function.

Successful PD requires prompt and safe insertion of the PD catheter into the peritoneal cavity. Various clinical specialists can insert a PD catheter using different techniques, such as open surgery, peritoneoscopy, percutaneously with a trocar, or the Seldinger technique. Interventional nephrologists are increasingly performing ultrasound-guided percutaneous insertion of PD catheters. The advantages of this technique are that it is relatively rapid, it can be performed in a procedure room, recovery is rapid, there is no need for general anesthesia, and the hospital stay is short (2–4). However, reports of pediatric percutaneous PD catheter insertion are rare. In this study, we present our single center experience using ultrasound-guided modified Seldinger insertion of Tenckhoff catheters compared with open surgical placement of catheters.

## Methods

### Patient Selection and Protocol

This single-center retrospective study of pediatric patients with end-stage renal disease (ESRD) examined 14 patients who received elective percutaneous PD catheter insertion from August 2018 to February 2021 and 10 patients (controls) who received PD catheter insertion by open surgery from 2015 to 2018. This study was approved by the Ethics Committee of Tongji Hospital, and each patient's father or mother provided written informed consent before the procedure. Patients were excluded if they were critically ill or had a history of PD-related fungal peritonitis.

The primary outcome was technical success rate. Technical success was defined as a functional catheter for at least 1 month after PD catheter insertion. The secondary outcome included 6-month catheter survival, length of incision, and potential early complications (bowel or bladder perforation, hemorrhage, pain, peri-catheter leakage, peritonitis, and exit-site/tunnel infection). Peritonitis was defined by at least two of the following outcomes: abdominal pain, with turbid PD fluid, with or without fever; white blood cell count in PD effluent >100 × 10^6^/L with more than 50% neutrophils; and growth of pathogenic microorganisms in effluent cultures.

### Catheter Placement Procedures

**Figure 1:**
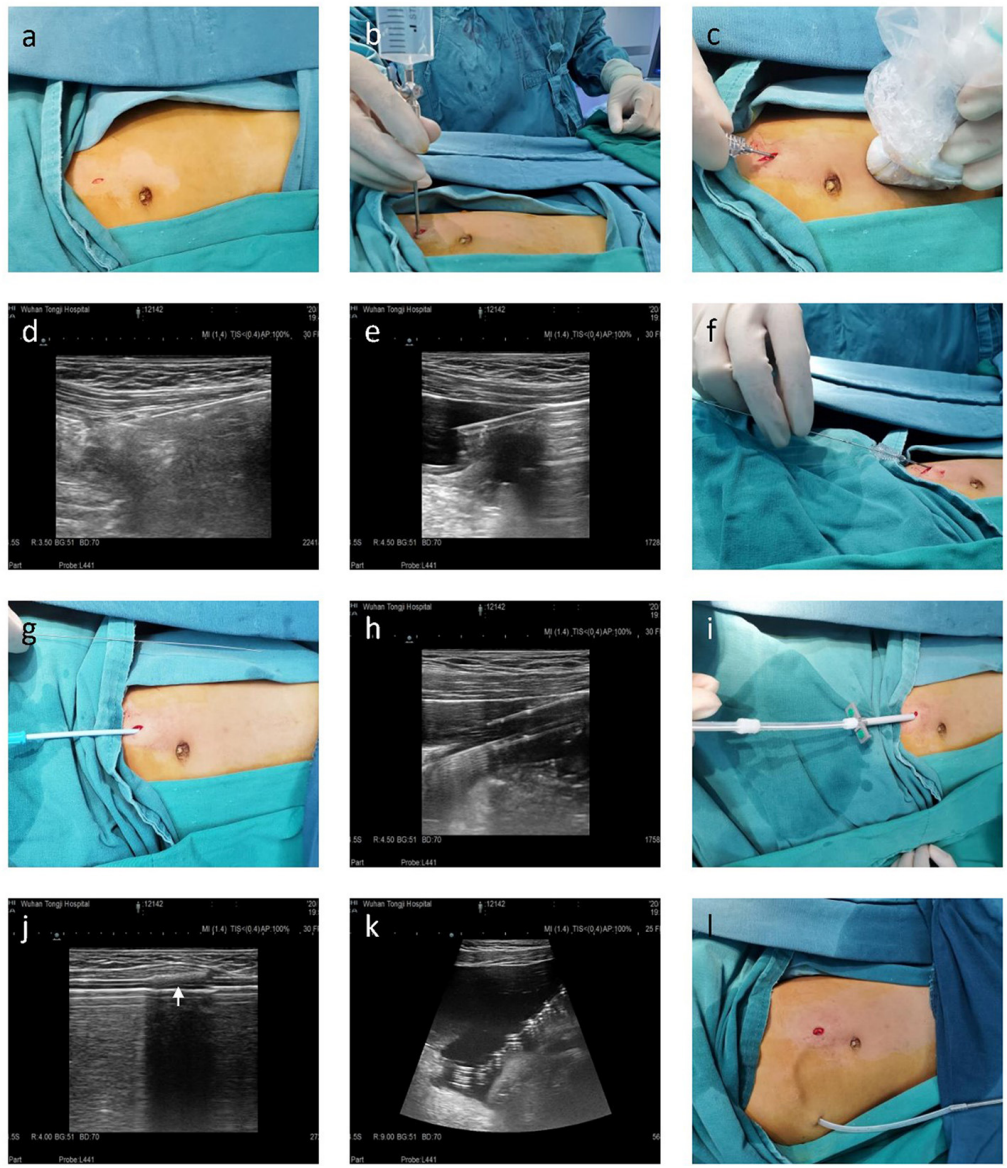
Seldinger placement of a Tenckhoff catheter in a representative pediatric patient in preparation for peritoneal dialysis. **(a)** A 1 cm linear incision was made, and subcutaneous tissue was blunt separated to reach the anterior sheath of the rectus abdominis. **(b)** A hydroperitoneum was established using a Veress needle. **(c–e)** A 18-gauge coaxial biopsy needle was advanced in a caudal direction toward the pelvis at 30–45° from the skin surface using ultrasound guidance. **(f)** A 0.035-inch guide wire was inserted through the coaxial biopsy needle and directed toward the pelvis. **(g,h)** 8-French, 12-French, and 14-French introducer sheaths were sequentially placed over the guide wire. **(i–k)** The PD catheter was introduced over the rigid guide wire within the 16-French pull-apart sheath after removal of the inner sheath dilator. Ultrasound was used to confirm the deep cuff and tip of the PD catheter reached the rectus abdominis muscle and Douglas pouch, respectively. **(l)** An 8–12 cm subcutaneous passage for the PD catheter was established using a stylet, and the second cuff was 2 cm from the exit.

For ultrasound-guided modified Seldinger placement of Tenckhoff catheters, the same pediatric nephrologist and sonologist inserted all PD catheters in an operating room (average time: 30 min). During each procedure, the patient was placed in a supine position, and peripheral intravenous access and routine monitoring were established. Before the procedure, the location of the catheter in the pelvis, the position of the deep cuff, and the exit site were marked. Then propofol was administered to achieve conscious sedation. The left quadrant of the abdominal wall was prepped and draped, and local anesthesia (xylocaine 1%) was administered from the entry site to the peritoneum. Then, a 1 cm linear incision was made at the puncture point, and the anterior sheath of the rectus abdominis was exposed by blunt dissection of subcutaneous tissue (Figure 1a). The sheath was punctured at the center using a Veress needle, and physiological saline (20 mL/kg) was introduced into the abdominal cavity, which moved away the greater omentum and parietal peritoneum (Figure 1b). After the establishment of hydroperitoneum, an ultrasonic probe that was within a disposable sterile endoscope sleeve was placed on the abdominal wall of the puncture site, and a sharp-tip 18 Gauge coaxial biopsy needle was rotated slowly through the rectus muscle at an oblique angle (30–45°) under ultrasound guidance to establish a trans-rectus abdominis tunnel of the appropriate length (Figures 1c,d). After entry into the parietal peritoneum, a blunt needle core was used to replace the sharp tip needle core, and insertion continued toward the vesicorectal fossa (Figure 1e). Upon entry to the lower pelvis, the needle core was extracted, and then a 0.035 inch guide wire was placed (Figure 1f). Over the guide wire, the insertion site was expanded gradually so that a 16-French pull-apart sheath could be inserted into the pelvic cavity (Figures 1g,h). The PD catheter with rigid guide wire was then passed through the sheath and placed deep into the pelvis, so that the deep cuff was directed into the rectus abdominis muscle (Figures 1i–k). The guide was then removed, and both parts of sheath were divided and also removed. The PD catheter was flushed with sufficient warm dialysis solution to confirm catheter function and no leakage or intra-abdominal bleeding. Then, an 4 to 8 cm subcutaneous passage for the PD catheter was established using a stylet, and the proximal end of the catheter was pulled through the exit and positioned so that the second cuff was 2 cm from the exit (Figure 1l). Finally, the incision was closed and the line was capped.

For open surgery, placement of the PD catheter was performed by pediatric surgeons who used the same procedure for most patients with general anesthesia (average time: 45 min). First, a left paramedian incision (3–4 cm) was established about 1–2 cm above the umbilicus. Then a small opening was established in the sheath of the rectus abdominis and peritoneum. The tip of PD catheter was introduced into the pelvic cavity using a stiff guide wire, and was then passed through a subcutaneous passage to the exit in the lower left quadrant. Finally, the peritoneum and sheath of the rectus abdominis were repaired using a vicryl suture, and the skin was closed using nylon stitches.

### Post-operative Treatment

After successful PD catheter insertion, a 1.5% PD solution containing low-dose heparin saline was used to rinse the abdominal cavity 3 times per day. The abdominal dressing was changed every 3 days until removal of the stitches, and exit-site care was performed daily. Automatic PD (APD) or continuous ambulatory PD (CAPD) was initiated at 3 days (percutaneous insertion) or 7 days (surgical insertion) after PD catheter placement, and the patients and families received training at this time using several low volume (up to 20 mL/kg) supine exchanges.

### Data Collection and Statistical Analysis

During the catheterization procedure, incision length, duration of the operation, and all intraoperative complications were recorded. After catheterization, pain, location of the PD catheter (confirmed by abdominal X-ray), drainage obstruction, and the color and clarity of PD effluent were recorded. The presence of complications within the first month and then every 3 months were recorded. These included infection at the PD catheter exit or tunnel, peritonitis, peri-catheter leakage and catheter displacement. These complications and catheter survival during 6 -months of follow-up were recorded.

Continuous variables are expressed as means ± standard deviations and were compared using Student's *t*-test or the Mann Whitney *U*-test. Categorical data were expressed as numbers and percentages and were compared using Fisher's exact test. A *P*-value below 0.05 was considered significant. All statistical analyses were performed using SPSS software package version 23.0.

## Results

**Figure 2:**
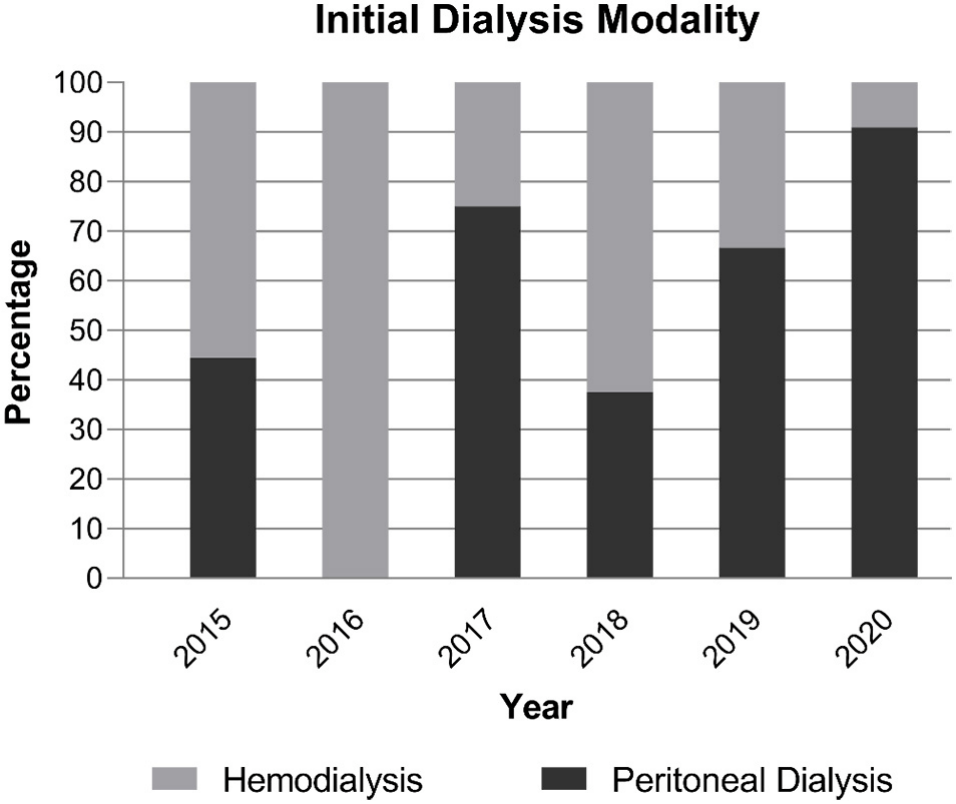
Initial dialysis modality used for pediatric patients in our center from 2015 to 2020.

We examined 14 patients who received PD catheters using ultrasound-guided percutaneous puncture with a Seldinger technique from August 2018 to February 2021. For comparison, we examined 10 patients who received open-surgical PD catheter insertion from 2015 to 2018. As shown in Figure 2, during the adoption of this technique in our center, an increasing percentage of new dialysis patients commenced PD rather than HD, and this percentage was 79% during 2019–2020 compared with 39% during the 4 previous years (*P* = 0.043).

As shown in Table 1, comparison of the two groups indicated they had similar mean body mass index (BMI: 16.91 ± 2.84 vs. 17.05 ± 2.11) and none of them were overweight. Nephrotic syndrome and congenital anomalies of the kidney and urinary tract were the common causes of ESRD in these patients. Only two patients had previous abdominal surgery (1 in the Seldinger group and 1 in the open surgical group). The patient in the Seldinger group previously received three open surgical PD catheter placements because of catheter migration or peritonitis, and had ultrasound findings indicating an impaired visceral slide test. Another patient in the open surgical group previously received laparoscopic right ureteral bladder replantation because of ureterovesical junction stenosis with hydronephrosis.

**Table 1:** Characteristics of patients in the two groups.

**Characteristic**	**Total (*n* = 24)**	**Seldinger group (*n* = 14)**	**Open surgery group (*n* = 10)**
Age, years ± SD	8.06 ± 4.21	7.55 ± 4.43	8.78 ± 3.76
Gender, M/F	12/12	7/7	5/5
BMI, kg/m^2^ ± SD	16.97 ± 2.56	16.91 ± 2.84	17.05 ± 2.11
History of hemodialysis, *n*	8	7	1
**Primary disease**, ***n***			
Nephrotic syndrome	8	3	5
CAKUT	4	2	2
Glomerulonephritis	3	2	1
Hemolytic uremic syndrome	2	1	1
Alport Syndrome	2	2	0
ANCA-associated vasculitis	2	2	0
Nephronophthisis	1	1	0
Unknown cause	1	0	1
Previous abdominal surgery, *n*	2	1	1

*ANCA, anti-neutrophil cytoplasmic antibody; BMI, body mass index; CAKUT, congenital anomaly of the kidney and urinary tract*.

Compared with open surgical placement, ultrasound-guided modified Seldinger placement was simpler, less invasive, and led to a faster postoperative recovery (operation time: 45 vs. 30 min; incision length: 4.4 vs. 1.1 cm). All 24 PD catheter insertions were technically successful, and the two groups had similar rates of achieving the primary endpoint. The 6-month catheter survival rates were 93% (13/14) and 90% (9/10) in the Seldinger group and open surgery group, respectively, 1 patient in each group experienced primary catheter dysfunction (Table 2). One patient in the Seldinger group with primary non-function experienced omentum wrapping and blockage at 1 month after surgery; this patient required laparoscopic omentum release and catheter replacement. One patient in the open surgery group with primary non-function experienced catheter migration; this patient required surgical reduction. Thus, ultrasound-guided modified Seldinger placement technique showed a similar catheter survival rate compared with open surgical placement (13/14 vs. 9/10 at 6 months).

**Table 2:** Operative characteristics and outcomes of catheter insertion in the two groups.

	**Seldinger**	**Open surgery**
	**group**	**group**
	**(*n* = 14)**	**(*n* = 10)**
Technical success, *n* (%)	14 (100%)	10 (100%)
Six-month catheter survival, *n* (%)	13 (93%)	9 (90%)
Primary catheter dysfunction, *n* (%)	1 (7%)	1 (10%)
Catheter migration	0	1
Omental wrapping	1	0
Length of incision, cm ± SD	1.11 ± 0.28^*^	4.40 ± 0.49
Hemorrhage, *n* (%)	1 (7%)	0
Pain, *n* (%)	3 (21%)^*^	7 (70%)
Catheter leak, *n* (%)	0	1 (10%)
Early infection, *n* (%)	0	0
Exit-site or tunnel infection	0	0
Peritonitis	0	0

* *Significantly different from the open surgery group, P < 0.05*.

We also examined other secondary outcomes (Table 2). Pain on flushing of the catheter occurred in 10 patients within 3 days of the procedure (7 in the open surgical group and 3 in the Seldinger group, *P* = 0.017). One patient in the Seldinger group had minor exit-site bleeding that resolved without intervention. Exit leakage occurred in 1 patient in the open surgery group, requiring surgical suture. No patients experienced bowel or bladder perforation, early peritonitis, or exit-site/ tunnel infection after 3, 7, or 14 days. One patient in the Seldinger group who previously had peritonitis developed peritonitis again at 5 months after recommencing PD, and another patient in the Seldinger group experienced inguinal hernia at 1 month after commencing PD.

Three of the 14 patients with percutaneous PD catheter insertion were on peritoneal dialysis at the end of the study period, 10 patients ceased PD because of successful renal transplantation, and 1 patient changed to HD at 1 year because of social factors.

## Discussion

Timely, accurate, and safe placement of a catheter is necessary for successful implementation of PD. The traditional techniques used for open and laparoscopic insertion of a PD catheter require availability of operating theater time and general anesthesia, which can impede timely catheter insertion and restrict access for patients with significant comorbidities. And laparoscopic catheter placement has been shown to have no superiority to open surgery (5, 6). In the present study, we present a modified Seldinger technique for placement of a Tenckhoff PD catheter. We demonstrated that ultrasound-guided insertion of a percutaneous PD catheter by a pediatric nephrologist, with the patient under mild sedation and local anesthesia, is less time-consuming, requires only a small incision, and enhances PD start. Although the incentive of health insurance policy, the spread of automated PD and the COVID-19 epidemic might influence the percentage of patients starting on PD. At our center, an average of 39% of pediatric patients per year initiated dialysis *via* PD from 2015 to 2018; this significantly increased to an average of 79% during the past 2 years (2019–2020).

Our two groups had no significant differences in age, gender, BMI, and cause of ESRD. It is worth mentioning that the modified percutaneous insertion technique was successful in one patient who had previous minor abdominal surgery. Although only one patient had complex abdomen, our study provides a “stepping stone” for future large studies that include these pediatric patients.

Nephrologists are increasingly interested in using percutaneous PD catheter insertion techniques, and several studies demonstrated efficacy and safety results that were similar to those from insertion by open surgery (3). However, it can be difficult to place a PD catheter accurately into the pelvis. Moreover, the conventional Seldinger technique, which uses a blind puncture process, has a risk of hollow-organ trauma and hemorrhage. Therefore, we modified the conventional technique by using ultrasound guidance and catheter fixation using a trans-rectus abdominis tunnel. The technical success rates of our Seldinger group and open surgery group were similar, and all 24 patients had successful drainage within 1 month. However, our use of color ultrasound guidance allowed rapid and accurate palcement of the catheter into the pelvis (operation time: 30 min; incision length: 1.0–1.5 cm), thus preventing organ injury and severe bleeding.

The duration of catheter survival after placement is an important consideration. Boujelbane et al. performed a meta-analysis and found no significant difference in the rate of 1-year catheter survival in adults who received percutaneous or surgical catheter insertion (3). Two registry-based studies from Italy of infants and pediatric patients (including young children under 2 years old and children aged 2–14 years) who received surgical PD catheter insertion reported the 1-year catheter survival rate was 70 & 78.1% and the 2-year survival rate was 51 & 58.5%, respectively (7, 8). These numbers are much lower than reported by Aksu et al. for children who received percutaneous PD catheter placement (92.4% at 1 year and 83% at 2 years) (9). We found that the 6-month catheter survival rates were similar in our Seldinger group and open surgery group (93 vs. 90%). Therefore, our ultrasound-guided modified Seldinger PD catheter insertion technique was at least as successful as open surgery after 6 months. Omental wrapping and catheter migration are the most common complications of PD catheter failure. Merrikhi et al. reported that omental wrapping occurred in 1 of 17 (5.9%) patients in their percutaneous insertion group, lower than in their open surgery group (10). Although our sample size was also small, the rate of omental wrapping in our Seldinger group was similar (7.1%, 1/14). We believe that our use of real-time ultrasound guidance facilitated this high rate of success. Similarly, the rate of catheter migration was lower in our Seldinger group than in our open surgery group, likely due to catheter fixation by establishing a trans-rectus abdominis tunnel of the appropriate length.

Catheter leakage and infections are complications that may occur when there is early use after placement of a PD catheter. Some guidelines therefore suggest waiting at least 2 weeks after placement before use (11). Two previous studies reported the incidence of catheter leakage was significantly greater in patients who received percutaneous insertion rather than insertion by open surgery (12, 13). We initiated PD at 3 days after catheterization in our Seldinger group, but none of the 14 patients experienced catheter leakage. This is likely due to the small incision, quick wound healing, the deep cuff of PD catheter insertion into the rectus abdominis muscle by blunt separation using 16-French peel-away sheath, and our initial use of low-volume supine exchanges. Infections, such as peritonitis and exit-site or tunnel infection, are another cause for revision of PD access (14). The 2012 ISPD consensus guidelines for the prevention and treatment of catheter-related infections and peritonitis in pediatric patients receiving peritoneal dialysis recommended that perioperative antibiotic prophylaxis be used within 60 min before the incision for PD catheter placement to reduce the incidence of early-onset peritonitis (15). However, we found that preoperative infection screening, bowel preparation, bathing with an antiseptic soap and strict sterile operation environment could reduce the risk of postoperative infections. Currently, we don't routinely give prophylactic antibiotics before surgery. Although prophylactic antibiotics are not given to all patients in our center, no patients experienced infection during the early postoperative period. Previous studies of percutaneous PD catheter insertion also reported very low rates of infections (13). In our cohort, one patient with a history of refractory PD-related peritonitis was complicated with peritonitis at 5 months after recommencing PD, suggesting that a history of refractory peritonitis might be a risk factor for recurrent peritonitis after PD access revision. Moreover, our use of a modified Seldinger insertion technique led to only 3 patients (21.4%) with postoperative pain, probably because of the small incision and no cutting of the rectus sheath and peritoneum. By contrast, 7 patients (70%) in our open surgery group reported abdominal pain.

This study has several limitations. First, this was a single-center study, we examined only 24 patients, and the follow-up period was only 6 months. A large-scale multicenter randomized controlled trial that compares PD catheter insertion techniques in pediatric patients is warranted. Second, this was a retrospective cohort study, and the results could have been affected by selection bias and observer bias. For instance, most of our patients had no history of previous abdominal surgery. Third, we did not compare the costs of percutaneous and surgical PD catheter insertion. Fourth, the insertion technique described here needs further improvements so it can be performed in patients with complex abdomen. For instance, it is impossible to conduct adhesion lysis with the Seldinger insertion technique described here.

In summary, ultrasound-guided modified Seldinger placement of a PD catheter, with the patient under mild sedation and local anesthesia, is safe and feasible for pediatric patients, including those who had previous minor abdominal surgery. Moreover, compared with an open surgical technique, this percutaneous insertion technique reduced the time of operation and hospitalization, and the incision was minimal.

## Data Availability Statement

The original contributions presented in the study are included in the article/supplementary material, further inquiries can be directed to the corresponding author/s.

## Ethics Statement

The studies involving human participants were reviewed and approved by the Ethics Committee of Tongji Hospital. Written informed consent to participate in this study was provided by the participants' legal guardian/next of kin. Written informed consent was obtained from the individual(s), and minor(s)' legal guardian/next of kin, for the publication of any potentially identifiable images or data included in this article.

## Author Contributions

YZ contributed to the study concepts and design. YYu, QX, YC, WH, PZ, SH, FY, YH, and YYi contributed to data acquisition. YYu, YZ, and JZ contributed to the data analyses and interpretation. YYu contributed to the statistical analysis. YYu and YZ contributed to the manuscript preparation, editing, and reviewing. All authors contributed to the article and approved the submitted version.

## Funding

This work was supported by the grants from the National Natural Science Foundation of China (Nos. 81570641 and 81770700).

## Conflict of Interest

The authors declare that the research was conducted in the absence of any commercial or financial relationships that could be construed as a potential conflict of interest.

## Publisher's Note

All claims expressed in this article are solely those of the authors and do not necessarily represent those of their affiliated organizations, or those of the publisher, the editors and the reviewers. Any product that may be evaluated in this article, or claim that may be made by its manufacturer, is not guaranteed or endorsed by the publisher.
